# Recent updates on central nervous system prophylaxis in patients with high-risk diffuse large B-cell lymphoma

**DOI:** 10.1186/s40164-023-00467-2

**Published:** 2024-01-03

**Authors:** Bernard Ji Guang Chua, Chen Ee Low, Chun En Yau, Ya Hwee Tan, Jianbang Chiang, Esther Wei Yin Chang, Jason Yongsheng Chan, Eileen Yi Ling Poon, Nagavalli Somasundaram, Mohamed Farid Bin Harunal Rashid, Miriam Tao, Soon Thye Lim, Valerie Shiwen Yang

**Affiliations:** 1 Division of Medical Oncology, National Cancer Centre Singapore, 11 Hospital Crescent, Singapore, 169610 Singapore; 2 Yong Loo Lin School of Medicine, National University of Singapore, 10 Medical Dr, Singapore, 117597 Singapore; 3 Duke-NUS Medical School, Oncology Academic Clinical Program, 8 College Road, Singapore, 169857 Singapore; 4 Translational Precision Oncology Lab, Institute of Molecular and Cell Biology (IMCB), 61 Biopolis Dr Proteos, Singapore, 138673 A*STAR Singapore

**Keywords:** Diffuse Large B-Cell Lymphoma, Non-Hodgkin’s Lymphoma, Central Nervous System, CNS Prophylaxis, Methotrexate

## Abstract

The use of central nervous system (CNS) prophylaxis for patients with diffuse large B-cell lymphoma (DLBCL) remains controversial. Although uncommon, CNS relapses are invariably fatal in this otherwise curable disease. Accurate identification of patients at risk and the optimal approach to CNS prophylaxis therefore remains an area of unmet need. The existing literature, largely retrospective in nature, provides mixed conclusions regarding the efficacy of CNS prophylaxis. The utility of CNS prophylaxis has itself been challenged. In this review, we dissect the issues which render the value of CNS prophylaxis uncertain. We first compare international clinical guidelines for CNS prophylaxis. We then interrogate the factors that should be used to identify high-risk patients accurately. We also explore how clinical patterns of CNS relapse have changed in the pre-rituximab and rituximab era. We then discuss the efficacy of CNS-directed approaches, intensification of systemic treatment and other novel approaches in CNS prophylaxis. Improved diagnostics for early detection of CNS relapses and newer therapeutics for CNS prophylaxis are areas of active investigation. In an area where prospective, randomized studies are impracticable and lacking, guidance for the use of CNS prophylaxis will depend on rigorous statistical review of retrospective data.

## Introduction

Central nervous system (CNS) relapses in diffuse large B-cell lymphoma (DLBCL) are uncommon, ranging from between two and ten percent of cases [1–3]. They are, however, invariably associated with poor outcomes with a guarded prognosis of two to six months [1–3]. The utilization of CNS prophylaxis in DLBCL has been largely extrapolated from the experience of treating high-grade lymphomas such as Burkitt’s lymphoma and acute lymphoblastic lymphoma (ALL). This practice remains widely used despite the lack of prospective randomized controlled trials to inform the value of CNS prophylaxis. Available data consists of largely retrospective studies and randomized prospective studies to inform on this issue have inherent practical challenges.

This review seeks to examine existing recommendations and guidelines, selection of high-risk patients for CNS prophylaxis, differences in the patterns of CNS relapses in the pre-rituximab and rituximab era, CNS-directed therapies, as well as the utility of intensification of systemic therapy and novel agents.

## Comparison of international clinical guidelines for CNS prophylaxis

Practices differ globally regarding the use of CNS prophylaxis in patients with DLBCL. A comparison of differences between selected published guidelines of different countries or regions is shown in Table 1.

**Table 1:** International Guidelines for CNS Prophylaxis in DLBCL

Guidelines	United States	Europe	United Kingdom	Spain	Australia
	NCCN [4] 2022	ESMO [5, 6] 2015, 2018	BSH [7] 2020	Spanish Lymphoma Group GELTAMO [8] 2017	Australasian Lymphoma Alliance [9] 2021
Strengths of Recommendations	All recommendations are of Category 2A	IPI Score and Extranodal Sites (II, A^a^) IV HD-MTX or IT MTX (IV, C)^a^	IPI Score, Extranodal Sites and Timing are of Category 1B Patients with testicular lymphoma should be considered for IT and systemic prophylaxis (Category 2B) All other recommendations are of Category 2C	IT MTX or triple IT (Category 2C) All other recommendations are of Category 2B	Intrathecal methotrexate prophylaxis is not recommended unless systemic prophylaxis is not deliverable (III-2, B)^b^ Two to three cycles of high-dose intravenous methotrexate (e.g. 3 g/m2) in addition to R-CHOP21 is reason-able in patients with high CNS-IPI and those with multiple or specific extranodal sites (III-2, C)^b^
Definition of high risk patients	CNS-IPI High-Risk (4–6)	IPI High-Intermediate (3) High (4–5)	CNS-IPI High-Risk (4–6)	CNS-IPI High-Risk (4–6)	CNS-IPI High-Risk (4–6)
Extranodal Sites	-–	> 1 Extranodal Sites	≥ 3 Extranodal Sites	> 1 Extranodal Sites (AND Raised LDH)	> 2 extranodal sites
	Kidney/Adrenal Testicular Breast Primary Cutaneous DLBCL, Leg-type	Kidney/Adrenal Testicular Breast Bone, Bone marrow	Kidney/Adrenal Testicular Intravascular Breast (Consider) Uterus (Consider)	Kidney/Adrenal Testicular Epidural Space Breast	Testicular Uterus Breast
Molecular	Translocations of MYC and BCL2 and/or BCL6	–	–	MYC rearrangements associated with BCL2 or BCL6 rearrangements	–
IV HD-MTX or IT MTX	IV HD-MTX and/or IT MTX	IV HD-MTX IT MTX -limited role	IV HD-MTX preferred If IV HD-MTX delivered successfully, no role for IT MTX IT MTX if IV HD-MTX cannot be delivered successfully	IV HD-MTX recommended IT MTX or Triple IT are reasonable options	IT MTX not recommended unless IV MTX not deliverable
Dose of IV HD-MTX	3–3.5 g/m2	–	At least 3 g/m2 Infusion time over 2-4 h	≥ 3 g/m2	3 g/m2
Number of Cycles	2 to 4	–	2 to 3	Alternate with Systemic Treatment	2 to 3
Timing	Intercalated or End of Treatment	-	As early as possible (Individualize intercalated or end of treatment approach according to patient)	Intercalated preferred (without delay of subsequent cycles)	Intercalated (risk of increased toxicity and delay in RCHOP delivery) or end of treatment
			If intercalated—Before Day 10 of RCHOP		

IT MTX dosing as per Spanish Group – 12 to 15 mg once per cycle for 4–6 cycles

Triple IT dosing as per Spanish Group – MTX 15 mg, Cytarabine 40 mg, Hydrocortisone 20 mg

*NCCN* National Comprehensive Cancer Network, *ESMO *European Society of Medical Oncology, *BSH* British Society of Haematology, *NSW* New South Wales, *IPI* International Prognostic Index, *CNS-IPI* Central Nervous System International Prognostic Index, *LDH* lactate dehydrogenase, *DLBCL* Diffuse large B-cell lymphoma, *IV HD-MTX* Intravenous High Dose Methotrexate, *IT MTX* Intrathecal Methotrexate, *IT* Intrathecal, *RCHOP* Chemotherapy regimen comprising of Rituximab, Cyclophosphamide, Doxorubicin, Vincristine, Prednisolone

^a^Adapted from the Infectious Diseases Society of America-United States Public Health Service Grading System [10]

^b^Adapted from the NHMRC evidence hierarchy

**Fig. 1:**
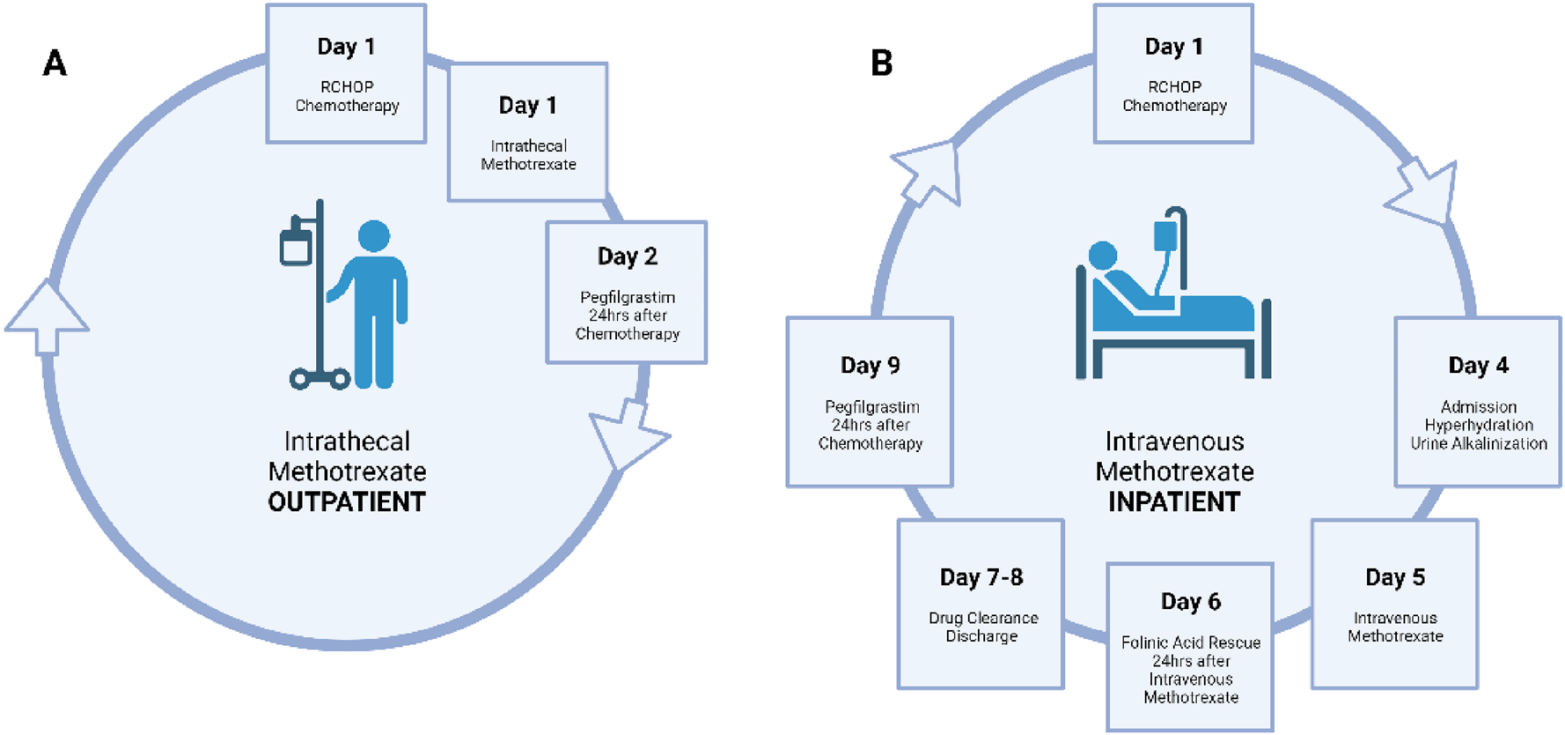
Typical treatment journey of patients receiving CNS Prophylaxis at the National Cancer Centre Singapore. **A** Patients receiving outpatient chemotherapy with intrathecal methotrexate administered via lumbar puncture in ambulatory setting. **B** Patients receiving outpatient chemotherapy and later being admitted for inpatient intravenous methotrexate. Patients remain admitted for pre-treatment hyperhydration and alkalinization as well as post-treatment folinic acid rescue and drug clearance. *Note that there may be variation in practices across different institutions around the world

While intravenous high-dose methotrexate (IV HD-MTX) appears to be a preferred option across various guidelines, it utilizes more resources given the need for inpatient monitoring, hydration, alkalinization of urine and drug clearance (Fig. 1). In settings where resources are limited or if patients are not fit for IV HD-MTX, intrathecal methotrexate (IT MTX) remains an alternative option [11].

## Which patients are at high risk of CNS relapses?

Given that the benefits, risks and side effects of CNS prophylaxis have not been well established in existing literature, it is important that patients with the highest risk of developing CNS disease are appropriately selected to maximize benefits and minimize harm [7].

### Prognostic models: CNS-IPI and more

Efforts to identify clinical and biochemical risk factors associated with CNS relapses have been well documented in the literature. Hollender et al. reported their findings in the pre-rituximab era based on a large group of high-grade Non-Hodgkin’s Lymphoma (Table 2). Factors such as number of extranodal sites, age, serum lactate dehydrogenase levels, serum albumin levels and presence of retroperitoneal lymph nodes were incorporated into this risk stratification model where high risk patients had CNS relapse risks of more than 25% [12].

**Table 2:** Prognostic Models in Predicting CNS Relapses for DLBCL Patients

Publication	No. of patients	% High Risk	Treatment	CNS Prophylaxis	Risk Factors	Risk Stratification	CNS Relapse Risk
Hollender 2002 [12]	1220 High-grade Non-Hodgkin's Lymphoma	12.30%	CHOP chemo or similar	12% received CNS prophylaxis IT and IV HD-MTX described	Extranodal Sites > 1 Elevated LDH Age > 60 Albumin < 3.5 mg/dL Retroperitoneal LN	Low (0–3) High (4–5)	5 year Risk Low: ≤ 6.2% High: ≥ 25%
Boehme 2009 [27]	306 aggressive B-cell Lymphoma	4.80%	R-CHOP-14	IT CNS Prophylaxis	Extranodal Site > 1 Elevated LDH Performance Status > 1	High (all 3 factors) Not-high (none)	2 year Risk Not High: 2.8% High: 33.5%
Schmitz 2016 DSHNHL [13]	1735 DLBCL	12.30%	R-Chemo	Not described	Extranodal Sites > 1 Elevated LDH Age > 60 Performance Status > 1 Stage III or IV disease Kidney or Adrenal Gland involvement	Low (0–1) Intermediate (2–3) High (4–6)	2 year Risk Low: 0.8% Intermediate: 2.9% High: 10.0%
Schmitz 2016 BCCA [13]	1597 DLBCL	22.90%	R-CHOP	Not described	Extranodal Sites > 1 Elevated LDH Age > 60 Performance Status > 1 Stage III or IV disease Kidney or Adrenal Gland involvement	Low (0–1) Intermediate (2–3) High (4–6)	2 year Risk Low: 0.8% Intermediate: 3.9% High: 12%
Kanemasa 2016 [28]	413 DLBCL	27%	R-CHOP or R-CHOP like regimens	15% received IT CNS prophylaxis 55% in high risk patients	Extranodal Sites > 1 Albumin < 3.2 mg/dL Stage III or IV disease Retroperitoneal LN	Low (0–2) High (3–4)	5 year Risk Low: 3.0% High: 26.4%
El-Galaly 2017 [29]	1532 DLBCL	9.50%	R-CHOP or R-CHOP like regimens	21% received CNS prophylaxis IT and/or IV HD-MTX and/or Cytarabine	Extranodal Sites > 2 on PET	Not-high (0–2 sites) High (> 2 sites)	3 year Risk Not High: 2.9% High: 15.3%
Tomita 2017 [14]	1220 DLBCL	19.10%	R-CHOP	No CNS Prophylaxis	Extranodal Sites > 1 Elevated LDH Age > 60 Performance Status > 1 Stage III or IV disease Breast and Testis considered high risk	Low (0–1) Low-Intermediate (2) High-Intermediate (3) High (4–5)	2 year Risk Low: 1.3% Low-Int: 4.6% High-Int: 8.8% High: 12.9%
Klanova 2019 GOYA [15]	1418 DLBCL	8%	R-CHOP or G-CHOP	9.9% received IT MTX and/or cytarabine	CNS-IPI - Low/Intermediate: 0 - High: 1 Cell of Origin - GCB: 0 - ABC/Unclassified: 1	Low: 0 Intermediate: 1 High: 2	2 year Risk Low: 0.5% Intermediate: 4.4% High: 15.2%

*DSHNHL* Deutsche Studiengruppe hochmaligne Non-Hodgkin-Lymphome, *BCCA* British Columbia Cancer Agency, *DLBCL* Diffuse large B cell lymphoma, CHOP *Cyclophosphamide, Doxorubicin, Vincristine & Prednisone*, *R-CHOP* Rituximab, Cyclophosphamide, Doxorubicin, Vincristine & Prednisone, *G-CHOP* Obinutuzumab, Cyclophosphamide, Doxorubicin, Vincristine & Prednisone, *CNS-IPI* CNS International Prognostic Index, *LDH* Lactate dehydrogenase, *IT MTX* Intrathecal methotrexate, *IV HD-MTX* Intravenous high-dose methotrexate, *GCB* Germinal center B-cell, *ABC* Activated B-cell

In the rituximab era, Schmitz and colleagues later developed the Central Nervous System – International Prognostic Index (CNS-IPI) score based on the German High-Grade Non-Hodgkin’s Lymphoma Study Group (DSHNHL) studies and validated it in an independent cohort of patients at the British Columbia Cancer Agency (BCCA) [13]. The CNS-IPI score consists of components in the IPI (International Prognostic Index) score (age, serum LDH levels, performance status, stage and extranodal sites) which were associated with worse outcomes in general, as well as kidneys and/or adrenal gland involvement. The validation study demonstrated that CNS-IPI is highly reproducible to estimate the risk of CNS relapse or progression in DLBCL patients treated with chemotherapy regimen comprising of rituximab, cyclophosphamide, doxorubicin, vincristine and prednisolone (RCHOP) chemotherapy. The two-year rates for development of CNS relapses between the DSHNHL cohort compared against the BCCA cohort were 0.6% vs 0.8% for low risk, 3.4% vs 3.9% for intermediate risk and 10.2% vs 12.0% for high risk [13]. However as the high-risk CNS-IPI group only consists of 55% of CNS relapses in the study cohort, other variables will need to be considered to further refine the selection of patients at high risk of CNS relapses. Importantly, high risk anatomical sites such as breast and testis involvement as well as consideration of cell of origin have a significant impact in prognosticating for CNS relapses [14, 15]. These will be discussed in subsequent sections.

### Anatomical locations

**Fig. 2:**
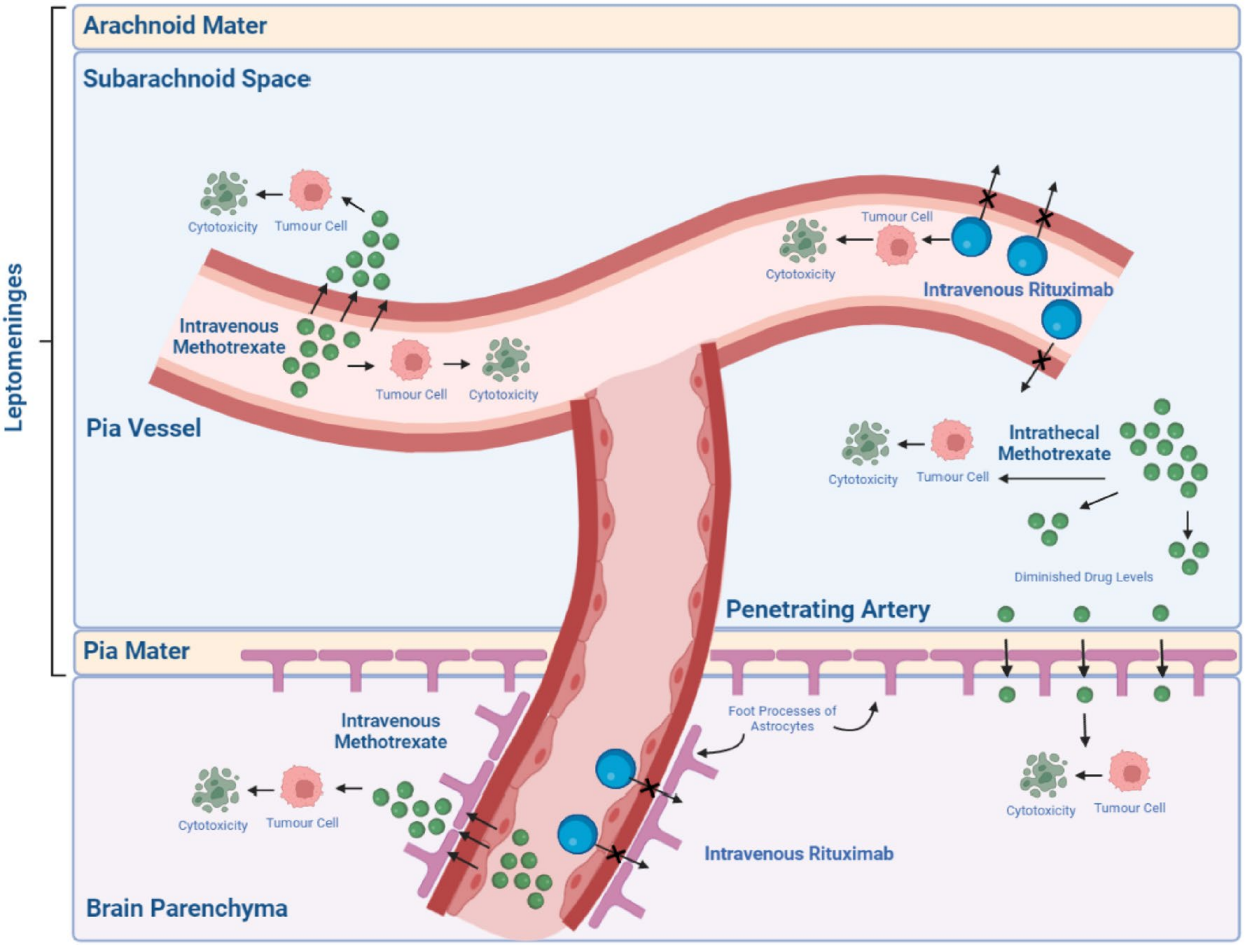
The blood–brain barrier and the effects of intravenous rituximab, intravenous methotrexate, and intrathecal methotrexate within it. Intravenous rituximab is a large molecule that is unable to cross from blood vessels to within the CSF in the subarachnoid space or into the brain parenchyma. However, they exert their cytotoxic effect within the blood supply in these areas and hence minimize CNS relapses. Intravenous methotrexate is a small molecule and therefore can cross the blood–brain barrier, exerting their cytotoxic effect within the brain parenchyma and to a certain extent within the CSF in the subarachnoid space as well. Intrathecal methotrexate enters the CSF within the subarachnoid space directly and may even cross the blood–brain barrier into the brain parenchyma. However, intrathecal administration does not guarantee uniformly consistent drug levels, especially when crossing through the blood–brain barrier, impairing the efficacy of its cytotoxic effect

Involvement of certain anatomical locations are also be associated with higher risks of CNS relapses for DLBCL patients. However, not all anatomical locations have been deemed equally high risk to warrant CNS prophylaxis. Kidney and adrenal gland involvement are most recognized and they are integrated into the widely utilized CNS-IPI score. Testicular, bone marrow and breast involvement are also given significant consideration for CNS prophylaxis and are included in recommendations by most guidelines as summarized in Tables 1 and 2 [4, 5, 7, 8, 16–19]. In a retrospective study by Tomita and colleagues, amongst patients with breast involvement, only 29% would have been considered high risk based on standard IPI score. In contrast, 89% of patients with adrenal and/or kidney involvement were at high-intermediate or high risk by standard IPI score [14]. In the most recent 5th edition of the World Health Organization Classification of Lymphoid Neoplasms, a new term of large Large B-cell lymphomas of immune-privileged sites was coined to distinguish primary B-cell lymphomas of the CNS, vitreoretina and testes in immunocompetent patient from other B-cell lymphomas [20]. This is to give recognition to distinct anatomical barriers, such as the blood–brain barrier (BBB) (Fig. 2), that render these sites an immune sanctuary. Primary B-cell lymphomas from these locations have unique immune regulatory systems resulting in differing pathogenesis as well as immunophenotypical and molecular features that may explain their CNS tropism. This will be discussed in a later section.

Anatomical sites such as the head and neck region (paranasal sinuses, hard palate), areas anatomically close to the CNS (epidural, dura, paravertebral, orbit) and others such as uterine and intravascular involvement have also been reported to have higher risk of CNS relapses [21]. However, these are not as consistently reported across studies, in part due to lower incidences, and hence highlights the importance of physician judgement when considering CNS prophylaxis.

### Cell of origin

Beyond the use of CNS-IPI score to risk stratify patients, other biomarkers have been evaluated to attempt to further improve the selection of patients at high risk of CNS relapse. Cell-of-origin (COO) subtype has a prognostic impact on the outcomes of DLBCL patients, with the activated B-cell (ABC) subtype or non-germinal centre subtype observed to have worse survival as compared to the germinal centre (GCB) subtype [15, 22]. ABC subtype DLBCL has also been characterized by gene alterations in the *CDKN2A* gene, affecting NF-κB signalling which may contribute towards CNS tropism [23, 24].

Savage and colleagues reported that ABC subtype DLBCL was associated with increased risk of CNS relapses [25]. This observation was later studied using data from the GOYA Phase III study investigating the efficacy of CD20 antibodies, obinutuzumab against rituximab, in DLBCL patients [26]. High CNS-IPI score and ABC or unclassified subtype DLBCL were independently associated with CNS relapsed (hazard ratios of 4.0; p = 0.02 and 5.2; p = 0.0004) respectively [15]. Combining both parameters in a proposed CNS-IPI-C score, high risk (defined by high CNS-IPI score and ABC/unclassified subtype) was associated with a two-year CNS relapse rate of 15.2% as opposed to 0.5% for low risk (defined by low CNS-IPI score and GCB subtype) [15]. However, considerations should be given when interpreting the results as due to the exclusion of the use of cytotoxic chemotherapy other than CHOP, there may be high risk patients who would have received IV HD-MTX as CNS prophylaxis that were not included in this analysis.

Therefore, risk assessment of CNS relapse for DLBCL patients should perhaps utilize the combination of CNS-IPI score and COO. Further validation studies in larger prospective cohorts are required to inform on this.

### Double-hit and triple-hit DLBCL

Double-hit or triple-hit lymphomas, where there are chromosomal translocations involving MYC, BCL2 and/or BCL6 oncogenes, are associated with a higher risk of CNS recurrence [30]. This group of patients represent about five percent of all newly diagnosed aggressive B-cell lymphomas [30]. In a retrospective analysis, cumulative incidence of CNS involvement was as high as 13% at three years [31]. Among those without documented CNS disease at diagnosis, eventual CNS involvement was lower in those receiving prophylactic intrathecal treatment than those without (5% vs 15% at three years, p = 0.017) [31]. Another analysis by Petrich et al*.* also showed that in patients without CNS involvement at diagnosis, methotrexate-containing CNS prophylaxis (intravenous or intrathecal) was associated with longer median overall survival (OS) than those without CNS prophylaxis (45 vs 14 months) [32]. This demonstrates that double-hit or triple-hit lymphomas are at increased risk of CNS recurrence and may benefit from CNS prophylaxis.

### Double-expressor DLBCL

In patients with double expressing lymphomas, where co-expression of MYC with BCL2 on immunohistochemistry are detected without associated translocations identified on in-situ hybridization, CNS recurrence risks can be as high as nine percent as compared to two percent in non-expressors [25]. This group of patients are more common than double-hit or triple-hit lymphomas, occurring in about one-third of DLBCL cases [25]. The increased risk of CNS relapses in double-expressor lymphomas appears limited to the ABC subtype of DLBCL as well as those with intermediate to high CNS-IPI scores [25, 30]. This shows that while double expression on immunohistochemistry can help identify a group of patients at higher risks of CNS relapses, risk stratification should also include other clinical and molecular factors.

### Genomic signature and CNS Tropism

The concept of CNS tropism that may be identified via biological markers is an ongoing area of research [33, 34]. *CDKN2A* and *ATM* deletions were identified as critical determinants of CNS tropism with in vivo mouse models [24]. NF-κB hyperactivation was also observed to promote CNS tropism [24]. Elevated levels of *ITGA10* and *PTEN* were observed to be associated with CNS relapses, whereas CD44 and cadherin-11 expression appeared to be protective [35].

Gene alterations in *BTG2, PIM1, DUSP2, ETV6* and *CXCR4* had been identified in more than 20% of patients with primary CNS lymphoma (PCNSL) in a study by Wang and colleagues [36]. When assessed in a group of patients with high-risk DLBCL who later developed secondary CNS recurrences, 70% were identified to have multiple alterations in these five genes [36].

Mutations in *MYD88*, *CD79B* and *PIM1* showed a predilection for extranodal sites, including the CNS and other anatomical sites where CNS prophylaxis is usually considered for, such as the breast and testes [37, 38]. These mutations are also noted to be almost exclusive to the ABC subtype of DLBCL, which has been considered a risk factor to be considered for CNS prophylaxis [15, 37].

While this will require further investigation and validation in larger cohorts, perhaps a genomic risk assessment with a selected genetic panel can be developed to identify patients who are at high risk of CNS relapses, warranting CNS prophylaxis. Further understanding of CNS tropism may also help with the future development of diagnostic tools and therapeutics to specifically target these pathways.

### Circulating DNA and clonotypic DNA

Existing evaluation techniques for CNS involvement primarily utilizes cerebrospinal fluid (CSF) sampling for both cytology and flow cytometry to detect CNS disease. However, episodic CSF sampling may not provide adequate yield to determine CNS involvement. CSF specimen may be contaminated with blood, impairing sample quality. CSF cytology has been shown to have a false negative rate of 20–60% [39]. Flow cytometry improves sensitivity for the detection of malignant cells in patients with negative cytology [40–42]. However, improved detection of disease in CSF specimen is still needed.

The use of circulating deoxyribonucleic acid (ctDNA) has gained traction in recent years. There may be value in detecting ctDNA within CSF to identify those that may be at high risk of CNS relapse. Bobillo and colleagues reported that presence of CSF ctDNA had been detected in the absence of radiological and flow cytometric findings of CSF disease prior to CNS relapse [40]. This suggests that ctDNA may facilitate earlier detection of CNS relapse and allow the provision of appropriate treatment in a timely manner.

Cerebrospinal fluid cell-free DNA (CSF cfDNA) was also reported to have a positive correlation with CNS-IPI score, with high concentrations observed in CNS-IPI high risk [4–6] group as compared to CNS-IPI low risk (0–3) group [36]. This suggests that presence of CSF cfDNA may be an indication of CNS involvement even if there are no clinical, radiological, or cytological evidence of disease.

As a larger proportion of CNS relapses occur mainly in the brain parenchyma in the rituximab era, CSF analysis may have limited utility in the detection of malignant cells as compared to those with either isolated or concurrent leptomeningeal involvement. Clonotypic, tumour-specific DNA rearrangements of the variable, diversity and joining (VDJ) regions of the immunoglobulin gene loci detected from tumour tissue derived genomic DNA can be used as a biomarker to detect CNS involvement in CSF samples via next-generation sequencing (NGS) based assays [43]. Of the patients who had brain parenchymal disease only, clonotypic DNA was identified even when they had negative CSF evaluation by cytology and flow cytometry [43]. Cumulative risk of CNS recurrence at 12 months from diagnosis was 29% in those who were positive as compared to zero percent in those who tested negative (p = 0.045) [43].

### Fluorine-18 fluorodeoxyglucose positron emission tomography/computed tomography

Fluorine-18 fluorodeoxyglucose (^18^FDG) positron emission tomography/computed tomography (PET-CT) is an important diagnostic tool for DLBCL patients. It demonstrates involved sites of disease and provides information regarding metabolic activity which can guide biopsy planning. It is also important in determining if bone marrow evaluation is required, as bone marrow evaluation is not commonly conducted upon negative PET-CT results. In a Korean study, authors investigated the value of pre-treatment ^18^FDG PET-CT in the prediction of CNS relapses after it was suggested that total lesion glycolysis (TLG) had prognostic value in DLBCL [44, 45]. Total lesion glycolysis is calculated by multiplication of mean standard uptake value (meanSUV) and metabolic tumour volume (MTV). Based on multivariate analysis, high TLG with a threshold margin of 50% (TLG50) was statistically significant as a prognostic factor to predict CNS relapses (p = 0.04) [44]. There was a significant difference in CNS progression free survival (PFS) between high and low TLG50 groups (43.9 vs 65.6 months) [44]. This shows that metabolic and volumetric parameters obtained from PET-CT imaging can provide further prognostic information for DLBCL patients and to identify those at high risk of CNS relapses.

A more recent study demonstrated that an artificial intelligence (AI) based model that incorporates clinical variables and imaging metrics from ^18^FDG PET-CT had improved prognostic utility as compared to models with clinical variables alone [46]. High-risk patients with this AI model had significantly increased risk of CNS relapse as compared to low-risk patients with hazard ratio of 5.42 [46]. When CNS-IPI score was combined with this AI model, high-risk patients had a two year CNS relapse probability of up to 17% [46]. This further shows the prognostic value of imaging parameters of PET-CT and the incorporation of AI to facilitate its use in clinical practice.

## Patterns of CNS relapse in the pre-rituximab and rituximab era

Patterns of CNS relapses have been observed to change after the advent of rituximab as a therapeutic agent for systemic treatment of DLBCL. Understanding these patterns can provide insights into developing new strategies to reduce the risks of CNS relapses in DLBCL patients.

### Locations of relapse: leptomeningeal vs parenchymal

In the pre-rituximab era, most relapses occur in the leptomeninges [12, 47, 48]. However, in the rituximab era, CNS relapses occur more commonly within the brain parenchyma, accounting for about 60% of all relapses as opposed to 15–20% for leptomeningeal relapses [49, 50]. Rituximab, an anti-CD20 monoclonal antibody is a large molecule with a molecular weight of 145 kilodaltons. Despite achieving high systemic dose levels when infused intravenously, its penetration into the CNS is dismal. CSF concentrations of rituximab reach about 0.1% of serum concentration when delivered at the standard dose of 375 mg/m^2^ [51]. This is largely due to the function of the BBB which does not only represent a single barrier between the blood–brain interface but consists of several levels of barriers occurring both in the macroscopic and microscopic levels.

Generally, only molecules of a low molecular weight of 400–600 daltons can cross the BBB at a microscopic level unlike rituximab [52]. Improved cytotoxicity with the addition of rituximab to cyclophosphamide, doxorubicin, vincristine and prednisolone (CHOP) chemotherapy may exert its effect on the vasculature within the leptomeninges where tumour cells also reside, leading to reduced leptomeningeal relapses. Molecular weight alone is unlikely to be the only factor contributing to passage of drugs across the BBB and many intricate interactions still unknown to us can affect this, such as the presence of efflux pumps [53]. As drug penetration across the BBB into the brain parenchyma reduces exponentially with increasing distance from blood vessels, the leptomeninges is exposed to high levels of systemically administered rituximab as compared to the brain parenchyma [54]. With a reduction in leptomeningeal relapses and the poor parenchymal penetrance of rituximab, the proportion of brain parenchymal relapses has in turn increased. Improved systemic control may accentuate this observed pattern and this also highlights the need for development of CNS penetrating drugs.

### Lack of benefit of IT MTX in the rituximab era

In the pre-rituximab era, the use of intrathecal chemotherapy for CNS prophylaxis in DLBCL patients was largely derived from the experience in Burkitt’s lymphoma and ALL which demonstrated mitigation of leptomeningeal CNS relapses. For Burkitt’s lymphoma, CNS prophylaxis with IT MTX containing regimens in the pre-rituximab era reduced rates of CNS relapses by about 6 to 11 percent [55, 56].

However, in the rituximab era, several studies have shown that the use of intrathecal chemotherapy did not result in reduced incidence of CNS relapses for patients with DLBCL [49, 57]. A reduced incidence of leptomeningeal relapses in the rituximab era, as explained above, may have also contributed to the difficulty in detecting a benefit. Methotrexate, one of the few drugs that can be administered intrathecally, has a low molecular weight of about 450 Daltons, allowing it to penetrate the BBB into the brain parenchyma. While intrathecal administration allows for good drug delivery within the CSF compartment, its efficacy remains dependent on CSF circulation for equal distribution within the CNS. For drugs administrated into the spinal subarachnoid space in the lumbar region, drug levels diminishes as it circulates rostrally, further minimizing its effect in the brain parenchyma [58, 59]. Whilst methotrexate is an attractive therapeutic option given its CNS penetrating properties, its efficacy in the treatment of DLBCL may not necessarily be comparable to other drugs such as rituximab [2].

The complex interface between blood, brain and CSF may contain further barriers for parenchymal penetration of drugs administered intrathecally through the CSF, with drug entry limited not just by diffusion but also efflux transporters and carrier-mediated transport systems [60, 61].

### Isolated CNS relapses versus concurrent CNS & systemic relapses

In the rituximab era, isolated CNS relapses in DLBCL patients account for about 70–75% of all CNS relapses with the remaining being concurrent CNS and systemic relapse [49, 62, 63]. In one study, there was no significant difference in the incidence of isolated CNS relapses between those who received RCHOP, CHOP or R-CHOEP (rituximab, cyclophosphamide, doxorubicin, vincristine, etoposide and prednisolone) chemotherapy [63]. There is nevertheless a numerically higher percentage of isolated CNS relapses in those who received rituximab-based chemotherapy as compared to CHOP without rituximab (74.1% vs 69.2%) [63]. This is also observed in the RICOVER-60 trial [27]. Collectively, this could indicate improved systemic control, as well as poor penetrance of rituximab into the CNS. This in turn gives further weight to the need to consider CNS prophylaxis with rituximab-based chemotherapy, to mitigate CNS relapses in this group of patients. As for patients with concurrent CNS and systemic relapses, it is likely that these patients constitute a group with poorer disease biology and worse prognosis overall. This group warrants further refinement of current selection criteria for poor risk disease and intensification of systemic therapy in the first line setting.

## Timing of CNS relapses: a true bimodal distribution?

CNS relapses have been reported in various studies to occur early, ranging between six to eight months from diagnosis of DLBCL [15]. However, there appears to be a bimodal distribution of early and late relapses. In one study, 24% of patients had early CNS relapses, including during initial systemic treatment, as compared to 76% of patients who had late CNS relapses that occurred about two years after diagnosis [64]. Bobillo et al*.* also demonstrated that CNS prophylaxis resulted in reduced CNS relapses at one year but this reduction was not statistically significant at five years [62]. These early relapses are therefore likely to represent occult CNS disease at baseline. Hence, administration of CNS prophylaxis appeared to reduce incidence of early CNS relapses when its true effect was early treatment of occult CNS disease.

In another study that looked at the impact of adding rituximab to CHOP chemotherapy on CNS relapses, the median interval from diagnosis of DLBCL to CNS relapse was similar at about six months [63]. These early relapses, which possibly represents undetected occult CNS disease, are unlikely to alter with the addition of rituximab which has poor CNS penetration. However, the median duration of survival after CNS relapses was significantly longer in those who received rituximab (365 days versus 75 days) which demonstrates that improved systemic control does improve survival outcomes even in the event of secondary CNS relapses [63]. Villa et al*.* showed that in patients who achieved a complete response, there was a marked reduction in CNS relapses [65]. These findings support the view that the addition of rituximab reduces late CNS relapses, possibly by improving systemic control.

## CNS-directed treatment

This section aims to compare the efficacy of CNS prophylaxis across the different routes by which CNS-directed treatment is given.

### IT MTX versus no CNS prophylaxis

The use of IT MTX as CNS prophylaxis in patients with DLBCL or aggressive B-cell lymphomas have been largely extrapolated from treatment regimens of Burkitt’s lymphoma and ALL where it is a standard component. However, its utility as CNS prophylaxis, especially in the rituximab era has been questioned.

A systematic review by Eyre et al*.* has shown that there are no existing published data that suggests a clear benefit of intrathecal chemotherapy alone (where methotrexate is the main component) as CNS prophylaxis in DLBCL patients undergoing anti-CD20 antibody and anthracycline based immunochemotherapy [49]. This is despite adjusting for confounding factors in a general population of DLBCL patients. Authors did qualify that the available evidence to suggest a genuine lack of benefit is also poor and therefore do not rule out its utility entirely [49]. Given the decreased proportion of CNS relapses occurring in the leptomeninges compared to brain parenchyma as described before, it is plausible that IT MTX as CNS prophylaxis has a diminished benefit in the rituximab era.

Should the role of IT MTX be considered as an alternative CNS prophylaxis, especially in patients who are contraindicated for IV HD-MTX? Such patients may include those with inadequate organ function and those experiencing significant myelosuppression. Elderly patients tend to have poorer organ function reserves and the utility of IT MTX in patients aged 70 years and above has also been reviewed separately [57]. In this study, no clear benefit for IT MTX was observed. Instead, an independent increased risk of infection related admission during systemic treatment was observed with the use of intrathecal CNS prophylaxis [57]. Whilst IT MTX remains a recommendation in some guidelines in those who cannot receive or tolerate IV HD-MTX, caution regarding its risks is to be exercised [7].

### IV HD-MTX versus no CNS prophylaxis

In the rituximab era, where there is a higher proportion of brain parenchymal CNS relapses compared to leptomeningeal, IT MTX is unlikely to provide significant intraparenchymal concentrations to achieve adequate CNS prophylaxis. IV HD-MTX therefore aims to provide better concentrations within the brain parenchyma. This has therefore been the preferred route of CNS prophylaxis across major guidelines, as shown earlier. However, is IV HD-MTX effective against CNS relapses? We examine the literature as follows.

In an Italian centre, CNS relapse rates were reported to be 12% in patients without IV HD-MTX as compared to 2.5% for patients who received prophylaxis (p = 0.03) [66]. However, this study has been critiqued for imbalances in the study population characteristics of advanced stage, raised LDH levels and high-risk anatomical locations. In another multicentre study by Ong et al*.*, three-year cumulative incidence of isolated CNS relapse was 14.6% in those who did not receive IV HD-MTX compared to 3.1% in those who did and this was statistically significant (p = 0.032) [67]. A further analysis in propensity score-matched patients also yielded similar results [67]. However, the relatively shorter follow-up time of 20 months for the cohort could potentially underestimate the number of late relapses.

On the other hand, Puckrin et al*.* demonstrated that there was no significant benefit of IV HD-MTX as CNS prophylaxis in a group of patients at high risk for CNS relapse treated in Alberta, Canada [68]. CNS relapse risk was 11.2% in those with IV HD-MTX as compared to 12.2% in those without. Even after accounting for confounding factors with multivariate analysis and propensity score analyses, there remained no association between IV HD-MTX and CNS relapse [68]. Another Korean study also demonstrated similar findings with the use of IV HD-MTX as CNS prophylaxis (12.4% vs 13.9%) [69]. The conclusions drawn remained the same after propensity score–matched and inverse probability treatment weighting (IPTW) analyses were conducted to overcome potential bias between the treatment groups.

Recently, Lewis et al*.* presented their findings of a large retrospective study of more than 2400 high-risk aggressive B-Cell lymphoma patients with more than five years of follow-up [70]. They found that for the study population, whilst there was a statistically significant reduction in 5-year risk of CNS progression from 8.5% to 6.9% (HR 0.59 p = 0.014), it was not a clinically meaningful reduction, translating to subjecting 63 patients to CNS prophylaxis in order to prevent one CNS relapse. This statistical significance was also not carried over when analysis was performed in patients that achieved complete response at the end of their frontline treatment, which is a group of patients where the direct effect of CNS prophylaxis with HD-MTX can be examined clearly after attaining good systemic control. A subgroup analysis also demonstrated no reduced risk of CNS progression in patients who received IT MTX, as compared to those who had no CNS prophylaxis (5-year CNS progression risk for IT MTX was 8.0% versus 8.5% in those with no CNS prophylaxis). There was also no difference between risk of CNS progression for patients who received intercalated HD-MTX and those who received it after systemic therapy. The authors accept the challenges in making firm conclusions from a retrospective study, however, recognition is due for their efforts to minimize bias by sound statistical methodology. Whilst this single study does not answer the question definitively, it is increasingly shifting the needle, amidst the body of literature currently available, towards the limited benefit of IV HD-MTX as CNS prophylaxis.

Given the above, the utility of IV HD-MTX in CNS prophylaxis has been cast into doubt. While we cannot exclude the possibility of definite benefit in a group of well selected patients with the highest risks of CNS relapses, it appears that the margin of benefit for most patients receiving IV HD-MTX as CNS prophylaxis is small if any. This may not be sufficient to justify the toxicities and burden imposed on the healthcare system. A randomized controlled trial remains the gold standard to conclusively demonstrate the efficacy of IV HD-MTX but this is inherently challenging to conduct given the low incidence of CNS relapses. Multicentre and multinational retrospective analyses of large populations may be a more realistic and practicable approach to guide treatment practices.

### IT MTX versus IV HD-MTX

Comparisons between intrathecal and intravenous routes of methotrexate CNS prophylaxis have largely been retrospective. In a single-centre study at the Memorial Sloan Kettering Cancer Centre, DLBCL patients who received RCHOP or RCHOP-like chemotherapy with high risk of CNS relapse determined by CNS-IPI score, high-risk anatomic locations and those with MYC and BCL2 rearrangements were reviewed [62]. Majority of patients (86%) received IT MTX. CNS relapse risk appears to be similar for both routes of administration with five-year CNS relapse risk of 5.6% for IT MTX as compared to 5.2% for IV HD-MTX [62].

Another multicentre study in the US that reviewed 1162 patients across 21 academic centres showed that the majority (77%) of patients received CNS prophylaxis intrathecally [59]. No significant differences in CNS relapses were observed between IT MTX and IV HD-MTX (5.4% vs 6.8% p = 0.40) [59]. Statistical strategies to account for differences between both groups such as propensity score-matching, adjustments for CNS-IPI, number of doses received, and backbone chemo regimen yielded similar findings. As death may represent a competing risk to CNS relapses, authors performed a competing risk analysis and no differences between route of administration was identified as well [64].

Current evidence in the literature suggests that there is no significant differences in CNS relapses between IT MTX and IV HD-MTX. However, this clinical question remains flawed and baseless when neither IT MTX or IV HD-MTX alone have proven significant benefit as CNS prophylaxis. Recommendations of IV HD-MTX in most guidelines are still based on observations of increased parenchymal relapses in the rituximab era as well as the theoretical understanding that IV HD-MTX may offer higher CNS penetration into brain parenchyma. IT MTX is considered only when IV HD-MTX is contraindicated. Even if the benefit of any CNS prophylaxis can be definitively proven, a study of the preferred route of administration would face at least the same, if not more challenges.

### Is the combination of IT MTX and IV HD-MTX required?

IT MTX can provide high CSF drug levels, exerting its effect within the leptomeninges mainly while IV HD-MTX can provide high brain parenchymal penetration. A combined approach has been suggested by Faqah et al*.* in their single-centre retrospective study in Pakistan [71]. Even though it was not statistically significant, patients who had received both IT MTX and IV HD-MTX appeared to have numerically lowest three-year CNS relapse rates and highest three-year overall survival rates when given with CHOP chemotherapy with or without rituximab [71]. Notably, in this study, rituximab was not mandated in view of drug access issues. Given that rituximab is now the standard of care in the treatment of DLBCL, omission of rituximab will result in suboptimal systemic control and lead to an associated increase in CNS relapses. Therefore, the combined use of both IT MTX and IV HD-MTX may appear more efficacious as it compensates for the poorer overall outcomes in those who did not receive rituximab. This is consistent with the notion that in the pre-rituximab era, combined IT MTX and IV HD-MTX was given as CNS prophylaxis, resulting in reduced CNS recurrences in higher risk patients (by IPI score) [72].

In primary testicular lymphoma, it involves an immunoprivileged site that is not unsimilar to that of the primary CNS lymphoma with many shared clinicopathological and biological features [73]. Hence it is not surprising that PTL have a higher CNS relapse risk of up to 30% [74]. For this group of patients at higher risk of CNS relapse, prophylaxis given with both IT MTX and IV HD-MTX did not offer adequate protection for the majority, suggesting that a combined approach may represent overtreatment without improved protective benefit [73]. However, a recent Phase II study [75] of 54 patients had shown that instead, a combination of intrathecal liposomal cytarabine and IV HD-MTX resulted in a 5-year CNS relapse rate of 0% as compared to 6% in a preceding trial [76] where only IT MTX was utilized.

Overall, risks of a combined approach, both in terms of drug toxicities and the additional procedural risks of a lumbar puncture, may not be justified when the utility of either IT MTX or IV HD-MTX is not clear.

### Logistics of IV HD-MTX administration

To overcome the challenges with administration of IV HD-MTX, some centres have shared their experiences in modifying how IV HD-MTX is routinely delivered. IV HD-MTX for CNS prophylaxis has been given either in between cycles of RCHOP chemotherapy (intercalated) or at the end of treatment. These practices vary across institutions. However, CNS relapses for DLBCL patients has been observed to occur early and reported to be within six to eight months from diagnosis [15]. Thus, giving CNS prophylaxis as early as possible by intercalating IV HD-MTX with RCHOP chemotherapy cycles has been postulated to provide timely protection against CNS relapses as opposed to end of treatment administration. Intercalating IV HD-MTX may increase toxicities during RCHOP cycles, such as myelosuppression and acute kidney injury (AKI), which will lead to delay in the delivery of systemic chemoimmunotherapy, compromising on treatment intensity which is not ideal. Wilson et al*.* conducted a retrospective multicentre study to compare these two approaches but no differences in CNS relapse incidence were noted [77]. However, there were increased toxicities and RCHOP delays with the intercalated approach [77].

If intercalation is chosen, it must be noted that patients who received IV HD-MTX more than ten days after RCHOP chemotherapy was associated with higher rates of treatment delays. It has also been shown that IV HD-MTX can be given as early as day 1 of RCHOP without delaying treatment schedules. Lower rates of neutropenic fever and AKI were observed when IV HD-MTX was initiated in Cycle 2 or later.

Administration of IV HD-MTX is often accompanied by the need for a hyperhydration regime with urine alkalinization as well as close monitoring of fluid balances and urine PH. They also require folinic acid rescue and plasma methotrexate clearance monitoring. Whilst patients receiving IV HD-MTX generally requires a hospital admission, there is an increased impetus to transition this to the ambulatory setting which has been described to be safe and feasible with appropriate patient selection.

### Cytarabine

#### Intravenous cytarabine

Cytarabine is a CNS penetrating agent and has been shown to be associated with low CNS relapse rates when incorporated to intensive chemoimmunotherapy regimen in high-risk DLBCL [78, 79]. When given at high doses of about 3 g/m2, cytotoxic levels of cytarabine can be achieved within the CNS. However, high dose cytarabine can only achieve a CSF concentration of about one percent of what would have been achieved with intrathecal cytarabine [80]. High dose cytarabine also has significant side effects such as myelosuppression, neurotoxicity, and ocular toxicity such as conjunctivitis. Its role as a CNS prophylactic agent remains to be investigated.

#### Intrathecal cytarabine

Cytarabine is also one of few drugs that can be administered intrathecally. Liposomal formulation of cytarabine allows for sustained CSF levels up to two weeks after administration, with similar anti-tumour activity and safety profile [21, 81, 82]. However, cytarabine is commonly administered as a triple-intrathecal combination consisting of methotrexate and steroids as well rather than in isolation [8]. This approach is adopted from established protocols in the treatment of ALL and Burkitt’s lymphoma. While it is a reasonable CNS prophylaxis for DLBCL patients, it has not been compared with IT MTX alone to demonstrate if addition of cytarabine reduces the incidence of CNS relapses. A prospective single-arm study showed that at a median follow-up of 40 months, no CNS relapses were observed in patients who received intrathecal liposomal cytarabine [83]. A retrospective study found that no CNS relapses were noted at a median followup of 3 years for a group of high-risk DLBCL patients who had received intrathecal liposomal cytarabine as CNS prophylaxis [84]. The use of intrathecal cytarabine in combination with IV HD-MTX may also be considered given the Phase II IELSG 30 study results of no occurrence of CNS relapses in 5 years in patients with primary testicular lymphoma who are associated with high risk of CNS relapses [75].

### Intrathecal rituximab

As discussed earlier, systemic administration of rituximab does not allow for significant CSF concentrations to be achieved due to its large molecular size. Hence, despite the significant improvements in survival outcomes, its role as an active CNS agent when administrated systemically is less impressive.

Comparatively, patients with active leptomeningeal disease may have disruptions in the BBB that may allow for higher CSF concentrations of rituximab when administered systemically, with CSF concentrations reported to increase to 3–4% of serum concentration [51, 85].

There have been case series and reports sharing that administering intrathecal rituximab showed promising efficacy in patients with CNS disease [86–89]. It has been reported that patients with both leptomeningeal and parenchymal involvement are most likely to progress even with combined intrathecal and intravenous rituximab, whereas those with isolated leptomeningeal disease had high complete remission rates of about 50% with combined intrathecal and intravenous rituximab [90].

We await further investigation regarding the use of intrathecal rituximab as CNS prophylaxis in DLBCL patients [NCT03688451].

## Intensification of systemic therapy to mitigate CNS disease

As previously discussed, effective systemic treatment for DLBCL is a fundamental factor in the prevention of CNS relapse. Here we discuss the specific agents that can be added to CHOP regimen and whether their addition can mitigate CNS disease.

### Etoposide

In the pre-rituximab era when CHOP chemotherapy was the standard of care, addition of etoposide was an independent prognostic factor for no CNS recurrence [91]. Risk of CNS recurrence was markedly reduced with cyclophosphamide, doxorubicin, vincristine, etoposide and prednisolone (CHOEP) chemotherapy with a relative risk ratio of 0.4 (p = 0.017) [91]. However, it is worth noting that this analysis also included patients with other histological subtypes such as Burkitt’s Lymphoma and other lymphoblastic lymphomas.

### Rituximab

Addition of rituximab to CHOP chemotherapy was also shown to reduce the incidence of CNS relapses from 6.9% to 4.1% [92]. In an analysis of patients from the RICOVER-60 trial, risk of CNS relapse was increased without IT MTX if they had not received rituximab [27]. However, if they had received rituximab, risk of CNS relapse was significantly lower with or without IT MTX [27].

### Rituximab and etoposide

Interestingly, intensification of chemotherapy with the addition of both rituximab and etoposide was not observed to have further decrease in CNS relapse rates. A single-centre retrospective study showed CNS relapse rate of 6.2% in rituximab, etoposide, prednisone, vincristine, cyclophosphamide and doxorubicin (R-EPOCH) group and 2.4% in RCHOP group although the difference was not significant (p = 0.301) [93]. The phase III CALGB 50303 trial observed CNS relapse rates of 4.0% in RCHOP group and 3.3% in R-EPOCH group [94]. These observations may indicate that there is a limitation to intensifying systemic treatment with multi-agent chemotherapy alone with regards to CNS control. Combining systemic and intrathecal chemotherapy approaches as well as other methods of achieving better systemic control could be more effective in reducing CNS relapse.

### ACVBP chemotherapy

Further intensification of chemotherapy with doxorubicin, cyclophosphamide, vindesine, bleomycin, prednisone (ACVBP) had improved event-free survival (EFS) and OS over standard CHOP chemotherapy in poor-risk aggressive non-Hodgkin’s lymphoma [48]. In this protocol, apart from induction ACVBP, it also includes a sequential consolidation therapy containing CNS active agents such as IV HD-MTX, etoposide, ifosfamide and cytarabine. Patients receiving the ACVBP protocol had lesser CNS progression or relapses compared to those receiving CHOP (RR 2.99; p = 0.002) [48]. In the rituximab era, R-ACVBP also had improved survival over R-CHOP in low-intermediate risk DLBCL [95]. No CNS relapses were noted in the R-ACVBP group as compared to two patients in the R-CHOP group [95]. A joint analysis of data from multi-center clinical trials showed that CNS relapse rates R-ACVBP arm was numerically lower than R-CHOP like regimen arm (1.6% vs 3.9%), although this was not statistically significant (HR2.4; 95% CI: 0.8–7.4; *p* = 0.118) [96].

### Intensive chemoimmunotherapy and autologous stem cell transplant

In a retrospective study by Puckrin et al*.*, patients who received consolidative autologous transplant or intensive chemoimmunotherapy regimens such as rituximab, cyclophosphamide, doxorubicin, vincristine and high-dose methotrexate (R-CODOX-M), rituximab, ifosfamide, etoposide and cytarabine (R-IVAC) or R-EPOCH demonstrated a trend towards lower risk of CNS relapse [68]. Rates of CNS relapse was 6.0% for both groups of patients compared to 14.6% in those receiving standard RCHOP (p = 0.09) [68]. These patients represent a high-risk group with more than 60% with CNS-IPI of 4–6 and more than 40% with double hit lymphoma [68]. Amongst patients with CNS relapses, 59% had concurrent systemic disease, which may suggests that most CNS relapses occur in the setting of inadequate systemic control [68]. Another retrospective study also observed a trend towards reduced CNS relapse risk with intensive chemoimmunotherapy regimens [97].

While these are promising results, its utility in the front-line setting remains controversial. Not only did upfront consolidation with HDC and autologous HSCT not improve survival as per a meta-analysis, a retrospective Japanese multicentre study also reported similar incidence of CNS relapses [98, 99]. A strong margin of benefit must be demonstrated in a randomized trial to justify the use of HDC and autologous HSCT in reducing CNS recurrence, especially given its toxicities and significant risks of mortality. Even if this is proven as an effective method of reducing CNS recurrence, its use also remains limited to younger and medically fit patients.

### CD19-directed chimeric antigen receptor (CAR)-T Cell therapy

CD19-directed chimeric antigen receptor (CAR)-T cell therapy has shown significant progress in recent years in the treatment of DLBCL with phase III studies showing EFS benefit of axicabtagene ciloleucel and lisocabtagene maraleucel in the second line setting over HDC and autologous HSCT [100, 101]. There were significant concerns with the inclusion of DLBCL patients with CNS involvement in view of the known adverse event of immune effector cell-associated neurotoxicity syndrome (ICANS) associated with CAR-T cell therapy. The mechanism of ICANS is postulated to be related to pericytes surrounding endothelial cells along capillary walls that form part of the BBB, which may also express an isoform of CD19 that is targeted by CAR-T cells [102]. However retrospective studies have reported that CAR-T cell therapy in patients with CNS relapses did not have an increase in ICANS and should not be excluded from these studies [103, 104]. It was also described in a patient that interleukin-6 levels had a sevenfold increase in the CSF as compared to matched serum samples [103]. In the ZUMA-12 study which explored the role of CAR-T cell therapy in the first line setting for high-risk patients, no CNS relapses were observed after a median follow-up of 16 months [105]. This approach holds promise as a CNS active therapeutic option and warrants further investigation.

## Other considerations

The CNS is a sanctuary site which many systemic anti-cancer agents are impermeable to. However, with the development of novel agents, with smaller molecular size, better therapeutic agents with CNS activity could become available. This may potentially alter the landscape of current approaches to CNS prophylaxis in patients with DLBCL. Case reports have shown responses to immunotherapy in DLBCL patients with CNS relapses either as monotherapy or in combination with rituximab [106, 107]. Other possible candidates for future research can include lenalidomide or Bruton’s tyrosine kinase (BTK) inhibitors. Lenalidomide is an immunomodulatory agent which can penetrate the BBB with CNS activity [108, 109]. CSF/plasma ratio of lenalidomide was observed to be as high as 50% and dose dependent increases in CSF penetration was noted [109]. Lenalidomide also showed single-agent activity in relapsed/refractory DLBCL and is well tolerated. Zanubrutinib, a second-generation BTK inhibitor, has been studied in DLBCL either as monotherapy or with RCHOP based regimen, with modest tumour responses [110, 111]. It was also demonstrated to have good penetration of the BBB with a mean CSF/plasma ratio by protein binding of 94.0% [112].

## Discussion

Beyond the possibilities of better detection of CNS disease and advancements in therapeutics, there remain other barriers to answer our clinical question.

Firstly, one major reason why there are mixed views regarding the role of CNS prophylaxis in DLBCL patients is the lack of a randomized controlled trial to investigate this clinical question. However, several practical challenges preclude the successful implementation of a well-powered trial, given the low incidence of CNS disease. For example, to detect a meaningful reduction of CNS disease from 10 to 6%, and therefore a number needed to treat (NNT) of 25, with an alpha of 0.05 and a power of 80%, between 1400–1500 high risk patients must be recruited to answer this question. Given that the CNS IPI the high risk group was only 12% of DLBCL [70], over 10,000 patients would need to be screened, a gargantuan effort that is nearly impossible to achieve. Thus, this clinical question may be more practically addressed by retrospective analysis of large datasets which may culminate into a meta-analysis to provide more generalizable conclusions after considering the relative weightage of each study.

Secondly, there are significant statistical challenges with regards to the analysis of existing evidence given the lack of randomized controlled trials. Existing data consists of largely retrospective studies where there are inherent differences in study population that are not controlled for. These imbalances in the study population preclude the ability to draw strong conclusions and only serve the purpose of identifying patterns for hypothesis generation. Propensity score matched analyses and IPTW allows for some adjustments of population characteristics to help to identify possible impact of the intervention. There can also be selection biases with retrospective studies. For example, when comparing between routes of administration of methotrexate, patients receiving IT MTX may inherently be frailer as they may have been unfit or unsuitable for IV HD-MTX.

Lastly, our clinical question is not only a scientific question but also an ethical one. While we decipher available evidence to determine the best timing of treatment, selection of patients or the best route of administration, the more fundamental question will be whether CNS prophylaxis is warranted at all. To answer this question in a study, a comparator arm of patients not receiving CNS prophylaxis will be required. This omission will need to be in a randomized fashion and not determined solely by physician or patient factors. This randomization will also include patients who are at high risk of CNS relapse. This may represent an ethical dilemma for physicians, as the omission of CNS prophylaxis in this group of patients may potentially represent a breach of his or her duty of care to the patient. However, we must also keep in mind that administration of CNS prophylaxis is not void of side effects such as nephrotoxicity and delays in systemic treatment. Even by applying the rule of “first do no harm”, it is unclear if the omission or administration of CNS prophylaxis represents harm.

## Future directions and conclusion

The available evidence in the literature, although largely retrospective in nature, increasingly suggests the lack of benefit of CNS prophylaxis in high-risk DLBCL patients. If any definite benefit can be proven, it is likely to be marginal and clinically insignificant to justify the associated risks for patients and accompanying healthcare costs. Further refinement of patient selection by molecular, histopathological and morphological characteristics may yet identify a group of patients that benefit from CNS prophylaxis. Development of efficacious CNS-penetrating therapeutics with fewer toxicities must also be pursued. Concurrent efforts should also be directed towards improving the detection of occult CNS disease in high-risk DLBCL patients that we would generally consider for CNS prophylaxis.

## Data Availability

Not applicable.
